# Synthesis, Characterization and Application of Advanced Antimicrobial Electrospun Polymers

**DOI:** 10.3390/polym16172443

**Published:** 2024-08-28

**Authors:** Maja Somogyi Škoc, Ernest Meštrović, Pierre-Alexis Mouthuy, Iva Rezić

**Affiliations:** 1 Faculty of Textile Technology, University of Zagreb, 10000 Zagreb, Croatia; maja.somogyi@ttf.hr; 2 Faculty of Chemical Engineering and Technology, University of Zagreb, 10000 Zagreb, Croatia; emestrov@fkit.unizg.hr; 3 Botnar Research Centre, Nuffield Department of Orthopaedics, Rheumatology and Musculoskeletal Sciences Medical Science Division, University of Oxford, Oxford OX3 7LD, UK

**Keywords:** synthesis, characterization, novel materials application, surface modification, electrospinning, antimicrobial resistance (AMR)

## Abstract

The aim of this work was to synthesize, characterize and apply advanced antimicrobial biocompatible electrospun polymers suitable for medical implants for surgical repairs. Injuries to the musculoskeletal system often necessitate surgical repair, but current treatments can still lead to high failure rates, such as 40% for the repair of rotator cuff tears. Therefore, there is an urgent need for the development of new biocompatible materials that can effectively support the repair of damaged tissues. Additionally, infections acquired during hospitalization, particularly those caused by antibiotic-resistant bacteria, result in more fatalities than AIDS, tuberculosis, and viral hepatitis combined. This underscores the critical necessity for the advancement of antimicrobial implants with specialized coatings capable of combating *Methicillin-resistant Staphylococcus aureus* (*MRSA*) and *Methicillin-sensitive Staphylococcus aureus* (*MSSA*), two strains notoriously known for their antibiotic resistance. Therefore, we developed an antimicrobial coating incorporating nanoparticle mixtures using the sol-gel process and applied it to electrospun polycaprolactone (PCL) filaments, followed by thorough characterization by using spectroscopic (FTIR, Raman, NMR) microscopic (SEM and SEM-EDX), and tensile test. The results have shown that the integration of electro-spinning technology for yarn production, coupled with surface modification techniques, holds significant potential for creating antimicrobial materials suitable for medical implants for surgical repairs.

## 1. Introduction

Antimicrobial resistance (AMR) is a pressing global health concern, jeopardizing the effectiveness of antimicrobial agents and posing formidable challenges to healthcare systems worldwide. Defined as the ability of microorganisms to withstand the effects of antimicrobial drugs, AMR threatens the prevention and treatment of infections, leading to increased morbidity, mortality, and healthcare costs [1]. Despite concerted efforts to address AMR, its prevalence continues to rise, underscoring the urgency of implementing comprehensive strategies to mitigate its impact and ensure the sustainability of antimicrobial use [2]. Antimicrobial agents, encompassing antibiotics, antivirals, antifungals, and antiparasitics, play a crucial role in combating infectious diseases in humans, animals, and plants. The development of AMR arises from the evolutionary capacity of microorganisms to adapt to antimicrobial agents through various mechanisms. Bacteria, viruses, fungi, and parasites can acquire resistance through genetic mutations, horizontal gene transfer, and selective pressure exerted by antimicrobial exposure [3]. Consequently, resistant strains emerge, rendering conventional treatments ineffective and limiting therapeutic options for infectious diseases.

The misuse and overuse of antimicrobial agents in human and animal population constitute primary drivers of AMR, accelerating the development and spread of resistant microorganisms. Inappropriate prescribing practices, self-medication, and non-adherence to treatment regimens contribute to the selection of resistant strains and undermine efforts to combat AMR [4]. Furthermore, inadequate infection prevention and control measures, coupled with poor sanitation and hygiene practices, facilitate the transmission of resistant pathogens, exacerbating the AMR crisis. In addition to this, a huge number of antibiotic compounds are emerging contaminants in the environment arising from animal and fish production and consumption [5].

The economic burden of AMR extends beyond healthcare expenditures, encompassing productivity losses, trade disruptions, and adverse effects on food security and agricultural sustainability [6,7]. Strategies to combat AMR include promoting antimicrobial stewardship programs, enhancing infection prevention and control measures, fostering research and development of new antimicrobial agents, and promoting global partnerships for coordinated action [8]. Furthermore, raising awareness among healthcare professionals, policymakers, and the public about the importance of prudent antimicrobial use and infection control practices is essential for mitigating the impact of AMR and preserving the effectiveness of antimicrobial agents for future generations [9]. The Sustainable Development Goals provide a framework for action, emphasizing the importance of multisectoral cooperation and sustained investment in combating AMR and ensuring the sustainability of antimicrobial use for generations to come [10,11].

Alongside the problem of AMR, there is an increasing number of biomaterials that are being proposed for implantation to support the repair of damaged tissues. This is, for instance, the case of electrospun implants, made of nano- or micro-fibers. Electrospinning is a technique widely used in various research fields, including tissue engineering and regenerative medicine, that enables the fabrication of nanofibrous scaffolds mimicking the extracellular matrix (ECM) of native tissues [12,13,14,15]. Electrospun materials and scaffolds possess unique properties such as a high surface-area-to-volume ratio, interconnected porous structure, and tunable mechanical properties, making them suitable candidates for biomedical applications, particularly in surgical reparation [16,17]. By closely resembling the native ECM of tissues, electrospun fibers facilitate cellular adhesion, proliferation, and differentiation [18]. This promotes tissue regeneration and enhances the integration of the scaffold with the surrounding tissue upon implantation. Additionally, electrospun scaffolds can be tailored to mimic the mechanical properties of specific tissues, providing mechanical support during the healing process [19,20,21].

Furthermore, electrospinning allows for the incorporation of bioactive molecules such as growth factors, cytokines, and antimicrobial agents into the resulting scaffolds. These bioactive molecules can promote tissue regeneration, angiogenesis, and immunomodulation, thereby enhancing the overall efficacy of the biological healing process [22,23]. Moreover, the sustained release of bioactive molecules from the electrospun scaffolds can provide prolonged therapeutic effects, leading to improved clinical outcomes [22]. In surgical repair applications, electrospun scaffolds have been explored for various tissue types, including bone, cartilage, skin, nerve, and vascular tissues. For example, fibers loaded with osteoinductive factors have been used to promote bone regeneration in orthopedic surgeries. Similarly, electrospun scaffolds functionalized with growth factors have shown promise in promoting the repair of cartilage defects in joint surgeries [23,24]. Additionally, electrospun nanofibrous scaffolds have been investigated for skin grafts, nerve conduits, and vascular grafts, demonstrating their versatility in diverse surgical applications [25,26].

Overall, electrospinning holds great potential in advancing surgical repair strategies by providing biomimetic scaffolds with tailored properties for tissue regeneration and functional restoration. Further research and development in this field are needed to optimize the design, fabrication, and clinical translation of electrospun nanofibrous scaffolds for effective surgical reparation [27].

In terms of materials, polycaprolactone (PCL) has gained significant traction as a biomaterial. Its versatility has led to its integration into a wide range of medical devices and therapies. For instance, electrospun PCL has been utilized in wound-healing applications, where its biocompatibility and controlled degradation make it suitable for promoting tissue regeneration [23].

Electrospinning is a versatile and widely used technique for producing ultrafine fibers from a wide range of polymers. The process involves applying a high voltage to a polymer solution or melt, which is then ejected through a small nozzle or spinneret. As the charged polymer jet travels towards a grounded collector, it undergoes stretching and thinning, leading to the formation of continuous fibers with diameters ranging from nanometers to micrometers. Electrospinning allows for precise control over fiber morphology and diameter, making it ideal for applications in various fields such as tissue engineering, drug delivery, filtration, and the creation of functional textiles. The resulting nanofibers possess high surface-area-to-volume ratios, tunable porosity, and unique mechanical properties, which can be further enhanced by incorporating functional additives, nanoparticles, or bioactive agents into the polymer solution prior to spinning. The repair of injuries affecting the musculoskeletal system that necessitate surgical intervention can still exhibit high failure rates. An example is the rotator cuff tendon tears, which show retears in about 40% following repair. This underscores the imperative for the development of novel materials capable of healing injured tissues more effectively.

**Figure 1:**
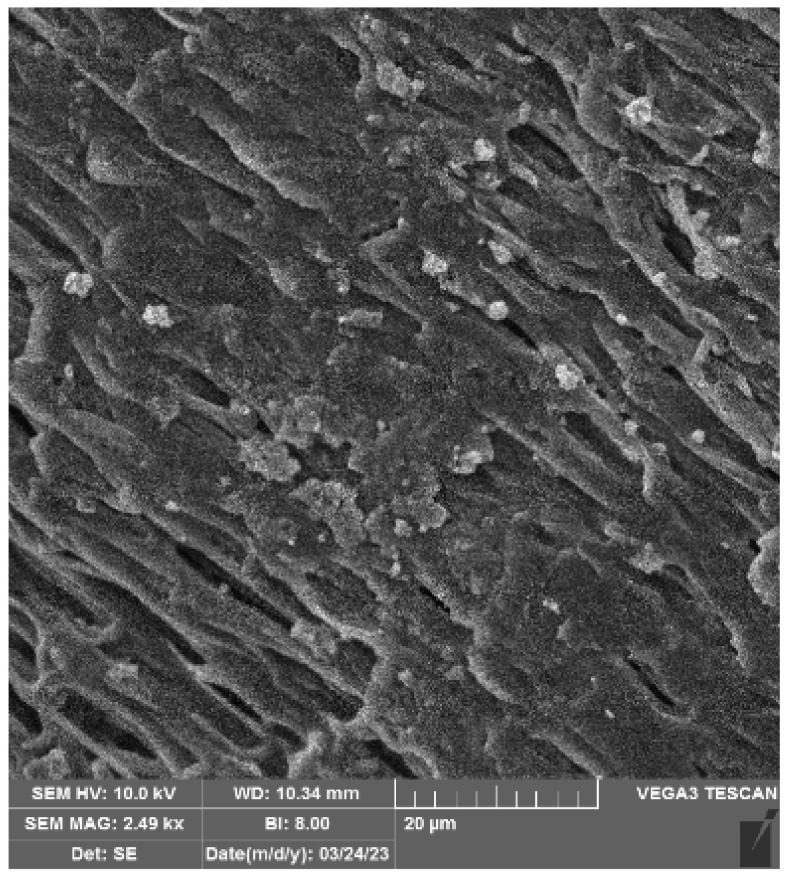
SEM micrograph of the yarn from electrospun fibers modified with nanoparticles.

Moreover, infections stemming from antibiotic-resistant bacteria during hospital care contribute to reduced healing rates [22]. These lead to more fatalities than the combined toll of AIDS, tuberculosis, and viral hepatitis [1]. Consequently, there is a pressing need for the creation of advanced antimicrobial implants featuring coatings that actively combat Methicillin-resistant Staphylococcus aureus (MRSA) and Methicillin-sensitive Staphylococcus aureus (MSSA)—microorganism strains known for their antibiotic resistance. The application of antimicrobial coatings, incorporating nanoparticle mixtures, through the sol-gel process onto material surfaces is a key strategy. These coatings undergo comprehensive characterization utilizing diverse spectroscopic, microscopic, and chromatographic methods. Combining this approach with the electrospinning of yarns and subsequent surface modification holds significant promise for generating antimicrobial materials suitable for medical implants. In this context, we propose the functionalization of continuous PCL yarns produced by electrospinning (Figure 1) through an antimicrobial coating comprising antimicrobial nanoparticles, employing the dip coating methodology. 

In our previous work, we presented and proposed the functionalization of woven and nonwoven polymer surfaces with different nanomaterials in order to achieve antimicrobial protection against *Methyl-Resistant Staphylococcus Aureus* (*MRSA*) and *Methyl-Sensitive Staphylococcus Aureus* (*MSSA*) [28,29,30,31], *Klebsiella pneumoniae* [32] and *Botrytis cinerea* [33]. The goal of this work was to apply these findings to electrospun yarns. To our knowledge, the modification of electrospun fibers by antimicrobially active gold nanoparticles has not been reported in the past. Such materials might find a broad range of applications in regenerative medicine.

Macroscopic images of the PCL electrospun yarns used in this research are shown in Figure 1. The materials were produced with the goal of promising applications in the development of medical devices targeted against drug-resistant bacteria [34,35,36]. In our previous investigation, we proved that electrospinning is a favorable process in which nanofibers of polylactone are produced in ranges of 250 to 500 nm [37]. 

The process of electrospinning was chosen as it stands out as one of the most effective approaches for generating polymer submicron fibers. In contrast to alternative methods, it offers the benefits of simplicity, versatility, and cost effectiveness. Such nanofibers have a wide variety of possible applications. Particularly in the medical field, electrospun fibers are scaffolds for tissue engineering, wound-healing devices, and drug delivery systems. Their efficacy as scaffolds for tissue engineering primarily stems from their ability to replicate the extracellular matrix that surrounds cells in tissues and organs. In addition, their application for repairing diverse biological tissues such as skin, tendon, ligament, bone, and cartilage is beneficial [37,38,39,40,41,42,43]. Micrograph of the yarn from electrospun fibers modified with nanoparticles obtained with SEM investigation is presented in Figure 1.

## 2. Materials and Methods

### 2.1. Electrospinning

The electrospinning process was performed through our previously developed methodology, as described by Mouthouy et al. [44]. The process started with the preparation of the electrospinning solution. Polycaprolactone (PCL), sourced from Sigma-Aldrich Chemical Company Ltd. in Dorset, UK, with a viscosity ranging between 1.5 and 2.2 dL g^−1^, was dissolved in 1,1,1,3,3,3-hexafluoroisopropanol (HFIP) obtained from Apollo Scientific Ltd. in Cheshire, UK. The continuous electrospun (ES) filaments were generated from the polymer solution using a single-nozzle electrospinning setup and a wire collector (100 μm in diameter, Goodfellow, Huntingdon, UK), following the procedure outlined previously [44]. The electrospinning process employed a high-voltage power supply system (30 kV, SL30P30/230, Spellman, West Sussex, UK) and a syringe pump from World Precision Instruments Limited, Sarasota, FL, USA. Both the nozzle and wire collector were situated within a glove box under continuous airflow to eliminate organic vapor emissions during operation. Before use, the wire collector was cleaned with 70% ethanol.

Maintaining a distance of 20 cm between the nozzle and wire collector, an average voltage of 8.6 kV was applied. The wire’s speed between the feeding and winding units was set at 0.5 mm s^−1^. Once the metal wire coated with the PCL material exited the glove box, the resulting ES fibrous mesh detached as a continuous filament, which was then wound onto a separate spool. At the conclusion of the process, the filament spool was removed and stored in a desiccator for subsequent stretching procedures.

### 2.2. Gold Nanoparticles

#### 2.2.1. Certified Reference Au Nanoparticles

The certified reference Au nanoparticles used in this research were 10 nm stabilized nanoparticles in suspension in 0.1 mM PBS, reactant free (752584 Sigma Aldrich product, batch number MKBX1673V) with the following certified properties: polydispersity index (PCI) < 0.2, core size 8–12 nm (average 10 nm), hydrodynamic diameter 11–25 nm, maximal absorption 522 nm, buffer for stabilization of 0.1 mM phosphate-buffered saline.

#### 2.2.2. Green Synthesized Au Nanoparticles

In our previous work, we developed and optimized a green synthetic method for producing gold nanoparticles using oxidoreductive enzymes. Additionally, a determination of optimal reaction conditions, reaction mechanism, and process scale-up was performed. Briefly, we applied enzymatic synthesis of gold nanoparticles by using several different oxidoreductive enzymes, namely, *Myriococcum thermophilum cellobiose dehydrogenase*, *Glomerella cingulata glucose dehydrogenase*, and *Aspergillus niger glucose oxidase*. The obtained Au NPs were characterized using UV–Vis spectroscopy and transmission electron microscopy (TEM). The surface plasmon resonance (SPR) absorption peaks of the Au-NPS synthesized using Mt CDH and Gc GDH were observed at 540 and 530 nm, revealing the synthesis of the Au-NPs with an average size around 60 and 40 nm. According to the image analysis performed on a TEM micrograph by counting 528 and 571 particles, the Au-NPs synthesized using Gc GDH after 24 and 48 h of the reaction have a spherical shape and a size distribution of 1.14–13.87 and 3.58–23.42 nm with an average size of 2.83 ± 2.40 and 6.63 ± 3.03 nm, respectively. After the initial synthesis, we monitored the Au NPs in time by TEM micrographs and observed that the nanoparticles were agglomerated and grew in size over time [45]. This was confirmed using nanoparticle tracking analysis, through which we observed agglomeration from sizes of 10 nm to 100 or even 200 nm. Therefore, the colloidally stabilized nanoparticles in the form of certified reference materials were used in antimicrobial screening of the antimicrobial efficiency.

### 2.3. Instrumentation, Protocols and Data

Samples were characterized by scanning electron microscope, coupled with spectroscopical screening (Raman, NMR), after which the modification of the yarns by the sol-gel procedure was performed by using a previously developed protocol [28].

Scanning electronic microscope “TESCAN VEGA TS5136LS” (Brno, Czech Republic) with the EDX detector was applied for the monitoring of the sample morphology and the determination of major sample components. The SEM-EDS instrumental parameters were as follows: working distance of 25 mm, energy 20 eV, and magnification 1000–10,000 times. The detectors used were the back scatter and the detector of secondary electrons, with a collection time of 30 s. The operating parameters of Sputter Coater SC 7620 applied for the coating of samples with a thin layer of Au/Pd were as follows: Power of 230 V (12 A), target Au/Pd, target distance 45 mm, power supply output 800 V, resolution 15 s, pump rate 25 L/min, sputtering rate 6 nm/min, coating thickness 1–20 nm, coating uniformity >10%, gas medium Ar.

The BRUKER EQUINOX 55 FT-IR/FT-RAMAN SPECTROMETER (Millerica, MA, USA) used was equipped with an interferometer equipped with Raman module FRA 106/S and Nd:YAG laser (1064 nm), spectral range for IR (NIR: 15,000–4000 cm^−1^; MIR: 4000–400 cm^−1^) and for Raman (3500–400 cm^−1^), and additional equipment: sample holders and cells for solid, liquid and gas samples, heater and cells for measurement of temperature-dependent spectra, equipment for measurements with attenuated total reflectance (ATR) and diffuse reflectance (DRIFT) techniques, and a fiber optic probe for in situ measurements.

The NMR produced by Bruker (Millerica, Massachusetts, USA) used was an NMR Spektrometar Bruker Avance (Millerica, MA, USA) III HD 400 MHz/54 mm Ascend with an AscendTM 9,4 T magnet, 5 mm inverse BBI probe with a Z-gradient and ATM unit (model PA BBI 1H/D-BB Z), 5 mm direct BBO CryoProbe Prodigy (model CPP BBO 400S1 BBH&F-D-O5 Z) with a Z-gradient, ATM unit and the associated equipment required for cooling the probe electronics with liquid nitrogen, temperature control system (VTU), BCU-I cooling unit, low-temperature measurement equipment and a SampleCase automation system.

The linear density of samples was measured out according to HRN ISO 2060:2008, Textiles—Yarn from packages—Determination of linear density (mass per unit length) by the skein method (ISO 2060:1994). The test parameters used during the determination of single-end breaking force and elongation at break using constant rate of extension were as follows: temperature 20 ± 2 °C, relative humidity 65 ± 4%, sample length 50 mm, clamp speed 500 mm/min, number of samples 10, and pretension according to linear density of samples.

### 2.4. Antimicrobial Testing

The antimicrobial activity of Au nanoparticles that were applied on electrospun fibers and yarns was assessed using the following model microorganisms: *Staphylococcus aureus*, *Escherichia coli*, and *Candida albicans* [29]. Firstly, the agar well diffusion assay was performed, followed by the serial microdilution broth assay. The agar well diffusion assay was conducted according to the guidelines set by the European Pharmacopoeia [46] and based on our previous research [29,32]. For the purpose of this investigation, inocula were prepared from fresh overnight bacterial cultures. Once the bacterial suspension was ready, sample solutions were applied to the wells in the agar plates in 50 µL volumes. Prior to incubation, the plates were pre-incubated at +4 °C for 1 h. This step was followed by incubation at +37 °C for 18 h under aerobic conditions, ensuring the environment remained dark throughout the process. The antimicrobial activity was then evaluated by measuring the diameters of the zones of growth inhibition around the wells (Tables 8 and 9).

## 3. Results and Discussion

Electrospun fibers, when used as wound dressings, offer several advantageous features. Their mesh-like structure provides high porosity, small pore size, and a large specific surface area. These characteristics serve as physical barriers against pathogens and facilitate the drainage of fluids from wounds. Consequently, these meshes can effectively prevent post-surgery adhesions [37]. Moreover, therapeutic and antimicrobial agents can be embedded within the fibers to improve the quality of healing and mitigate the risk of infection.

Through meticulous monitoring of process variables and optimization of reagent chemical composition, elestrospun fibers can be functionalized with active surface coatings effective against antibiotic-resistant microbial strains [35]. In this work, the results of functionalization of electrospun polylactone fibers with gold nanoparticles will be presented.

### 3.1. Characterization of Precursor before Modification of Electrospun Fibers with Au Nanoparticles by Deep Coating (Sol-Gel) Procedure

**Figure 2:**
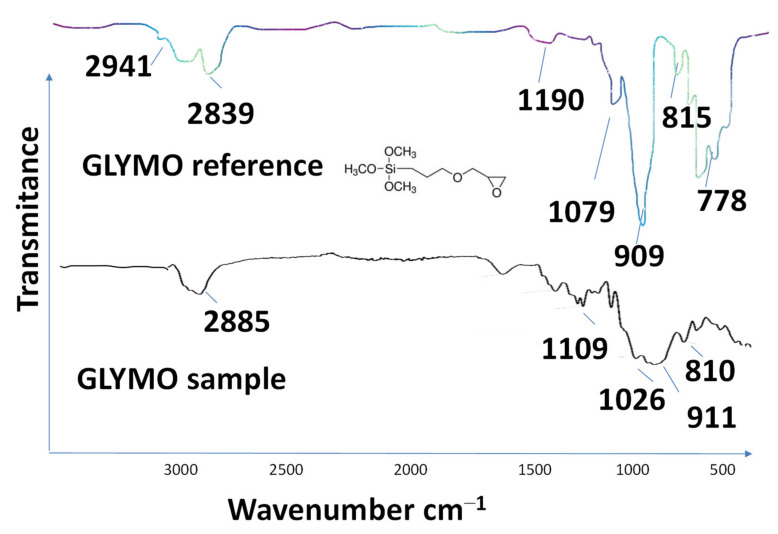
FTIR results of the precursor GLYMO obtained after the analysis of reagent used in this research (bottom, thin black line) and in comparison, with the FTIR recorded in the literature spectra (top, thick colored line) [39].

**Figure 3:**
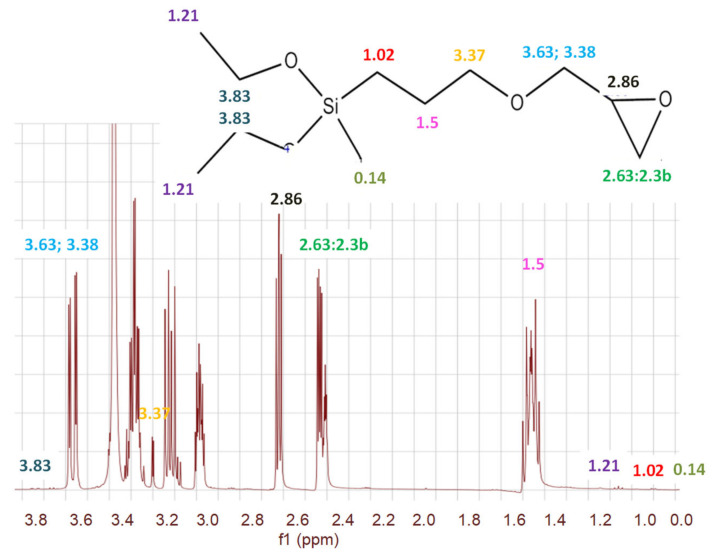
^1^H nuclear magnetic resonance spectroscopy (1H NMR) spectra at 400 MHz of pure GLYMO precursor prepared for electrospun sample. 1H–13C coupling is observed, peaks c* (δ = 3.83 ppm) and c** (δ = 3.38 ppm), which increase with the number of repeating units. Functional group labels refer to those in the chemical structure shown in the bottom right corner according to the predicted model [40,41,42]. The NMR spectrum of GLYMO (glycidyloxypropyltrimethoxysilane) typically shows characteristic peaks corresponding to the methoxy groups (-OCH3) attached to silicon, the epoxy group, and the propyl linker chain. The chemical shifts for the methoxy groups usually appear around 3.5 ppm, while the signals for the epoxy group protons can be observed between 2.5 and 3.2 ppm. Additionally, the propyl chain protons contribute to peaks in the region of 0.5 to 1.5 ppm.

**Figure 4:**
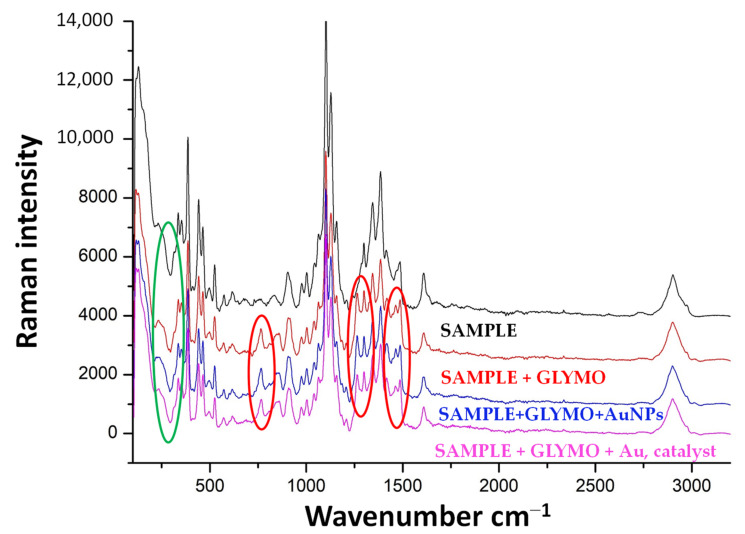
Raman spectra 500–3500 cm^−1^ (sample = black line, sample + GLYMO = blue line, sample + GLYMO + Au NPs = red line, sample + GLYMO + Au/ZnO NPs = pink line) with characteristic peaks at 1359, 1588 and 1595.8.

**Figure 5:**
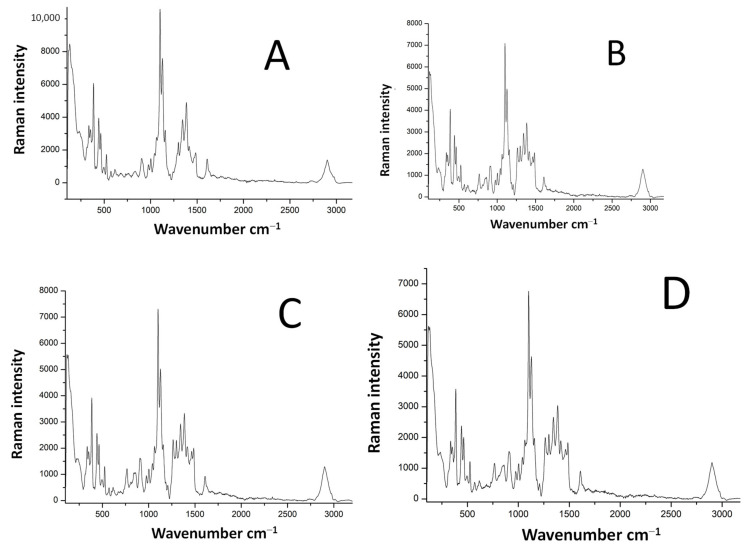
Separated Raman spectra 500–3500 cm^−1^ ((**A**)—untreated sample, (**B**)—sample + GLYMO, (**C**)—sample + GLYMO + Au NPs, (**D**)—sample + GLYMO + Au/ZnO NPs).

We employed FTIR (Figure 2), NMR (Figure 3) and Raman spectroscopy (Figure 4 and Figure 5), techniques to determine the structure and functional composition of the sample and of the precursor GLYMO utilized in the sol-gel textile functionalization experiments. The step of characterization of the degree of purity significantly impacts the modification efficiency, and any unwanted impurities may lead to alterations in the functionality of sol-gel process, resulting in changes in the functional groups observed in the spectra of samples before and after modification.

As can be seen from Figure 2 and Figure 3, the target molecule of investigation was GLYMO. FTIR spectra of GLYMO (3-(2,3-Epoxypropoxy) -propyltrimethoxy-silane, or Glycidyl 3-(trimethoxysilyl)-propyl ether) showed the most important peaks as follows: the peak from the epoxide asymmetric C-O-C stretch that appears from810 to 950 was recorded at 910 cm^−1^. Epoxides are characterized by a three-membered ring composed of two oxygen atoms and one carbon atom. Their spectra typically display three peaks, corresponding to the stretching and contracting of bonds within this ring, occurring within the ranges of 1280–1230, 950–810, and 880–750. Notably, the latter two peaks are particularly prominent. Monitoring the magnitude of these peaks enables tracking of the processes of an epoxy resin modification. The GLYMO spectrum was identified by the Si-O-C bands at 1200–1000 cm^−1^ and the oxirane ring peaks at 910 cm^−1^ (also at 3047). Aside from the oxirane ring, the Si-O-C bands were studied at 1109 cm^−1^.

The symmetric C-O-C stretch, where the two C-O bonds stretch while the C-C bond contracts, was found at 810 cm^−1^. Other peaks were recorded at 778 cm^−1^ originating from C-H; a strong, sharp peak at 1024.9 cm^−1^ is related to C-O stretching/O-H deformation; the peak at 1075 cm^−1^ originates from C-C stretching; 1109 cm^−1^ C-O stretching at C3; 2839 cm^−1^ is linked to CH of CH_3_, CH_2_, CH and peaks at 2885 cm^−1^ and 2941 cm^−1^ are linked to CH_3_ aliphatic CH; an absorption band at 2912 cm^−1^ to 2986 cm^−1^ is a characteristic of C-H stretching. In addition, the original spectra contained a broad band at 3200–3485 cm^−1^ characteristic of O-H stretching arising from the intermolecular and intramolecular hydrogen bonds. Both the reference and the sample used in modification had the same peaks that are related to the characteristic functional groups. It is important to acknowledge that variations in the quality of precursor materials can exert a notable influence on the performance of the end product [36].

The band around 1600 cm⁻^1^ in the FTIR spectrum of the GLYMO reagent most likely occurs due to the bending vibrations of adsorbed water (H-O-H bending). GLYMO contains methoxy groups that can hydrolyze to form silanol groups, which readily adsorb water from the environment. This is common for silane coupling agents exposed to ambient conditions where moisture can be present. Hydroxyl groups resulting from partial hydrolysis of methoxy groups could contribute to this band as well.

Proper analysis would also consider other bands in the FTIR spectrum to confirm this assignment and ensure no other functionalities contribute to this absorption. Other possible assignments for the 1600 cm⁻^1^ band in GLYMO could be as follows: C=C stretching of aromatic rings or conjugated systems, which is less likely for pure GLYMO, which does not inherently contain aromatic rings. Secondly, in the presence of amine functional groups or in a case that the GLYMO has reacted with compounds introducing amine groups, bending vibrations of NH_2_ groups could appear around this region. However, in the pure reagent, this is also very unlikely. Therefore, the assumption was that the absorbance of water molecules occurred and caused this change in the FTIR spectra.

In our previous research, we found GLYMO to be much better than TEOS and TMOS, particularly when the suspension is carefully homogenized by sonication prior to deep coating. In addition, the purity of the reagents was shown to cause diverse effects in the efficiency of deep coating. Therefore, in order to confirm the assumption of the purity of the GLYMO, the NMR of the pure reagent [40,41,42] was recorded and the result is presented in Figure 3. It would be more efficient to introduce Au NPs directly during the fabrication of the scaffolds (during their reduction from HAuCl4 or electrochemical deposition), rather than using commercially available nanoparticles, which are prone to agglomeration during storage. However, in this work, we are only developing a green synthetic methodology for producing Au NPs and this part is explained further in the text.

### 3.2. Characterization of Electrospun Fibers before and after Modification

Raman spectra, captured before and after modification (Figure 4 and Figure 5), revealed newly formed bonds in the samples, indicating that the textile carrier experienced minimal alteration, with no significant formation of new chemical bonds during the sol-gel functionalization process. Figure 5A–C shows the Raman spectra of the unmodified sample, and samples modified with GLYMO, GLYMO-Au NPs and GLYMO-Au NPs with a catalyst. A broad peak marked with a green-colored circle and centered at around 300 cm^−1^ suggests the presence of a SiO_2_ shell. Interesting changes in the spectra of the samples before and after the modification are marked with red circles, and as can be seen from the spectra, newly formed linkages were formed: epoxy groups before modification around ~905 and 910 cm^−1^, and after the modification the area around 1100 cm^−1^ can be linked to Si–O groups, (particularly Si–O–C and Si–O–Si bridges). In addition, there is a conversion of methoxy groups in precursor GLYMO, which can be monitored at ~2870 cm^−1^, and from this, it can be concluded that the Raman spectra of samples before and after the functionalization have significant differences. However, there was no difference in the resulting spectra of samples modified with and without a catalyst.

Acidic catalysts, such as HCl, influenced the sol-gel process significantly [43], as hydrolysis resulted in a homogeneous hybrid structure. The presence of acid also decreased the thermal stability of the hybrid materials. In the proposed reaction, the precursor GLYMO reacted in reactions of hydrolysis and condensation, in which alkoxide groups and epoxy rings opened. Therefore, it can be concluded that this precursor was a good starting point for creating hybrid materials with covalent bonds between the organic and inorganic phases. Moreover, the kind of network formed during the polymerization process strongly depended on the type of the catalyst used in the reaction: this modified the reactivity of the organic moiety of the GLYMO and a glass-forming behavior was obtained only when the epoxy ring of the GLYMO was cleaved.

### 3.3. Results of Density, Breaking Force and Elongation at Break

**Figure 6:**
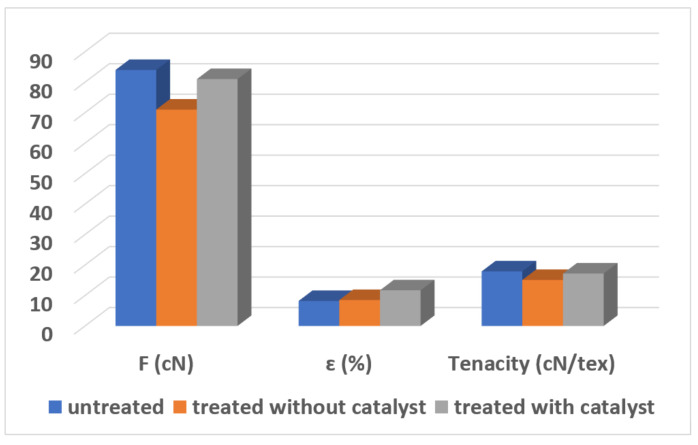
Determination of single-end breaking force and elongation at break using constant rate of extension (CRE) tester.

The obtained results are presented in Table 1, Table 2, Table 3 and Table 4, and in Figure 6.

**Table 1:** Results of linear density of samples carried out according to HRN ISO 2060:2008 “*Textiles—Yarn from packages—Determination of linear density (mass per unit length) by the skein method (ISO 2060:1994)*” [47,48].

Code	Tt [dtex]
1 P	4.7
2 P	5.2
3 P	5.7

**Table 2:** Results of breaking force and elongation at break—sample 1 P.

Treatment		1P Untreated		II A—I P Gold Nanoparticles without Catalysts		II B—I P Gold Nanoparticles with Catalysts	
n/ parameters		F [cN]	ɛ [%]	F [cN]	ɛ [%]	F [cN]	ɛ [%]
n	1	67	8.1	50	5.4	94	16.5
	2	69	8.7	72	12.3	82	15.0
	3	87	12.9	80	9.9	88	11.1
	4	95	8.4	73	8.4	86	13.2
	5	94	9.6	70	6.0	83	13.2
	6	90	8.4	44	0.3	80	10.8
	7	88	6.0	65	8.4	75	9.0
	8	83	7.8	69	6.0	89	12.6
	9	82	6.3	76	8.1	79	11.7
	10	84	6.3	71	10.2	82	15.3
$\bar{X}$		84	8.2	67	7.5	84	12.8
σ		10	2.0	11	3.3	6	2.3
CV [%]		11.5	24.4	16.9	44.2	6.7	17.9

**Table 3:** Results of breaking force and elongation at break—sample 2 P.

Treatment		2P Untreated		II A—2 P Gold Nanoparticles without Catalysts		Gold II B—2 P Nanoparticles with Catalysts	
n/ parameters		F [cN]	ɛ [%]	F [cN]	ɛ [%]	F [cN]	ɛ [%]
n	1	75	6.9	63	8.4	87	15.0
	2	87	6.3	42	7.2	77	12.6
	3	61	2.7	66	12.6	73	16.2
	4	48	3.9	67	9.6	71	15.9
	5	52	6.0	65	7.8	60	15.9
	6	54	4.5	76	7.2	67	12.9
	7	44	4.5	56	16.5	69	11.7
	8	46	9.0	74	9.0	55	11.7
	9	55	5.4	56	31.8	64	14.4
	10	66	5.1	/	/	66	9.9
$\bar{X}$		59	5.4	63	12.2	69	13.6
σ		14	1.8	10	7.9	9	2.2
CV [%]		23.3	32.3	16.5	64.9	13.2	15.9

**Table 4:** Results of breaking force and elongation at break—sample 3 P.

Treatment		Untreated		II A—3 P Gold Nanoparticles without Catalysts		II B—3 P Gold Nanoparticles with Catalysts	
n/ parameters		F [cN]	ɛ [%]	F [cN]	ɛ [%]	F [cN]	ɛ [%]
n	1	37	0.3	42	4.8	55	6.6
	2	51	4.8	53	6.3	30	2.7
	3	55	6.0	49	7.2	49	5.1
	4	57	10.2	45	7.2	40	0.0
	5	58	6.9	33	3.9	72	9.3
	6	54	5.4	30	0.3	25	3.3
	7	46	3.6	/	/	38	0.3
	8	37	6.3	/	/	49	5.1
	9	34	3.9	/	/	37	4.5
	10	44	4.5	/	/	53	6.0
$\bar{X}$		47	5.2	42	5.0	45	4.3
σ		9	2.6	9	2.6	13	2.8
CV [%]		18.6	49.2	20.9	53.2	29.9	66.2

The influence of acidic catalysts, exemplified by HCl, on the sol-gel process is profound, as evidenced by hydrolysis, leading to the formation of a homogeneous hybrid structure [43]. However, the presence of acid has been found to decrease the thermal stability of these hybrid materials. In the proposed reaction, the precursor GLYMO undergoes hydrolysis and condensation reactions, causing alkoxide groups and epoxy rings to open. This process suggests that GLYMO serves as a promising starting point for the creation of hybrid materials with covalent bonds between the organic and inorganic phases. Additionally, the type of catalyst employed significantly influences the network formation during the polymerization process. Specifically, the reactivity of the organic moiety of GLYMO is modulated by the catalyst, leading to the manifestation of glass-forming behavior only when the epoxy ring of GLYMO undergoes cleavage.

This research work is a part of our project ABBAMEDICA that is focused on the sol-gel modification of different surfaces. We successfully modified woven and nonwoven medical textiles, 3D printed biodegradable polymers for medical devices, and electrospun materials (presented in this manuscript). Therefore, our focus was not on modifying the scaffolds during their production, which might result in better mechanical effects, but on the effects of the sol-gel process. However, it has to be noted that the mechanical tests, as well as the SEM micrographs, show that the morphology of the scaffolds was worse after sol-gel modification; so, in future work, another experimental design (such as modifications in the scaffolds during electrospinning) could be more recommendable.

### 3.4. Results of SEM Investigation of Samples before and after the Modification

**Figure 7:**
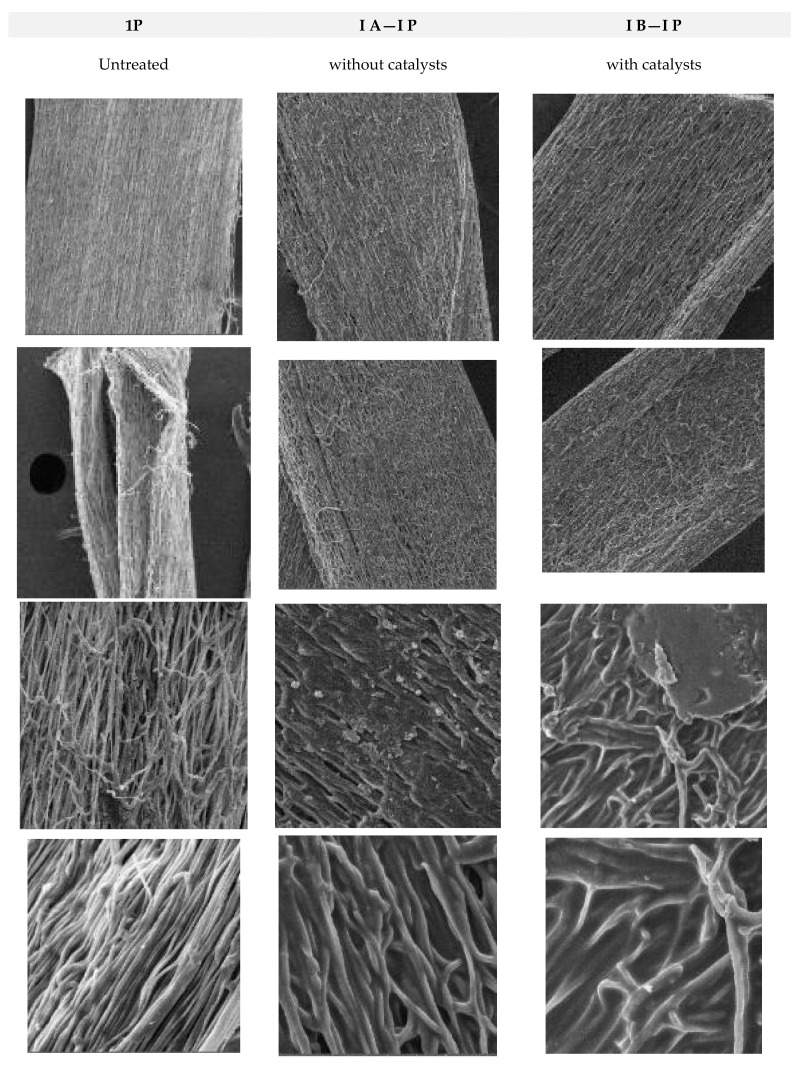

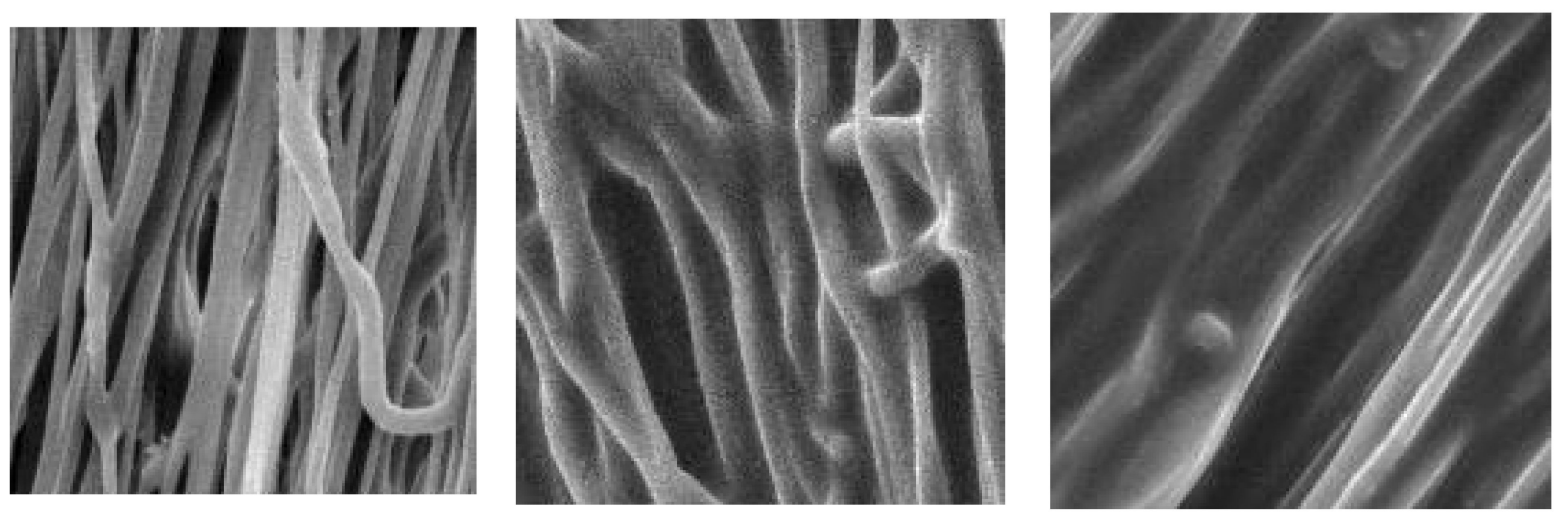
SEM images recorded of electrospun fibers before and after modifications. The first column shown non-treated yarns, and the second and the third show the modified yarns with nanoparticles, without applied catalyst and with HCl acid used as catalyst for faster modification.

**Figure 8:**
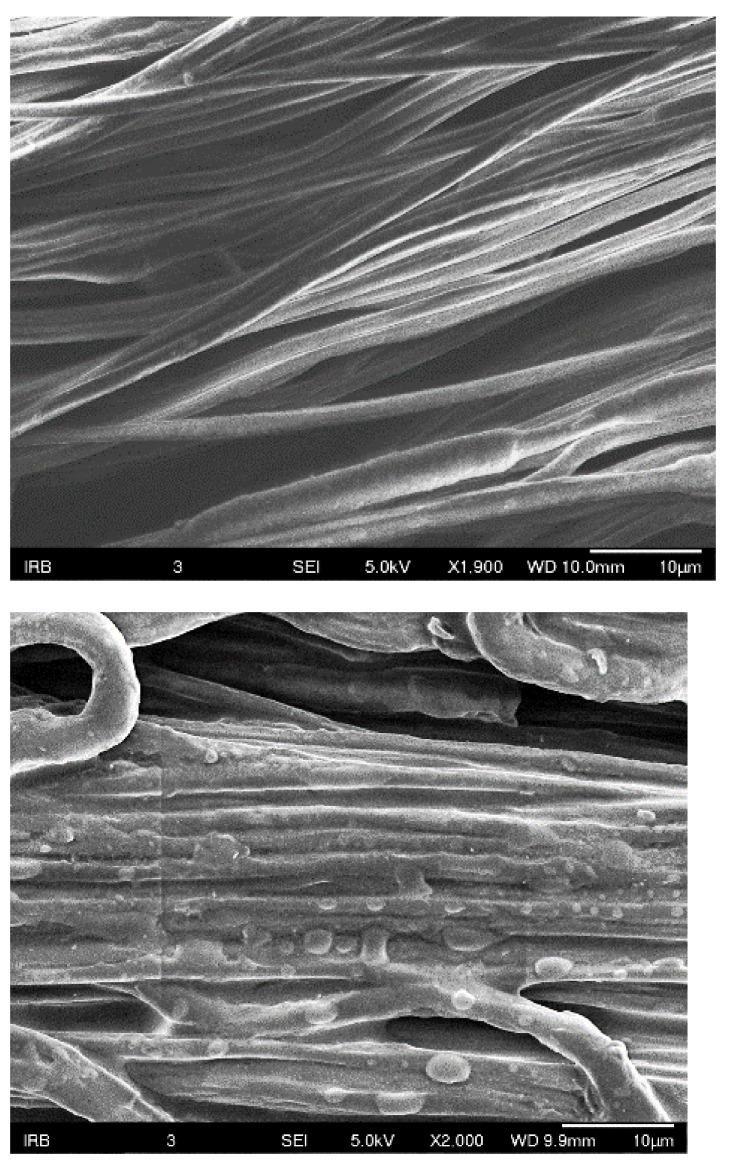
SEM images recorded of electrospun fibers before and after modifications. The first column shown non-treated yarns, and the second and the third show the modified yarns with nanoparticles, without applied catalyst and with HCl acid used as catalyst for faster modification.

The SEM micrographs presented in Figure 7 and Figure 8 of the surface of the composite electrospun yarn sample reveal that a smooth thin film was obtained on the substrate containing Au nanoparticles. In addition, the coating was evenly and homogenously distributed over the surface. Thus, it can be concluded that the advantages of the sol-gel method applied as a deep coating technology enable homogeneity and ease of composition control, and a low processing temperature enables an even distribution of the films on the substrate.

#### 3.4.1. Results of EDX Investigation of Samples after the Modification with Catalyst Recorded by Bruker Nano GmbH Berlin, Germany (SEM Tescan Vega)

**Figure 9:**
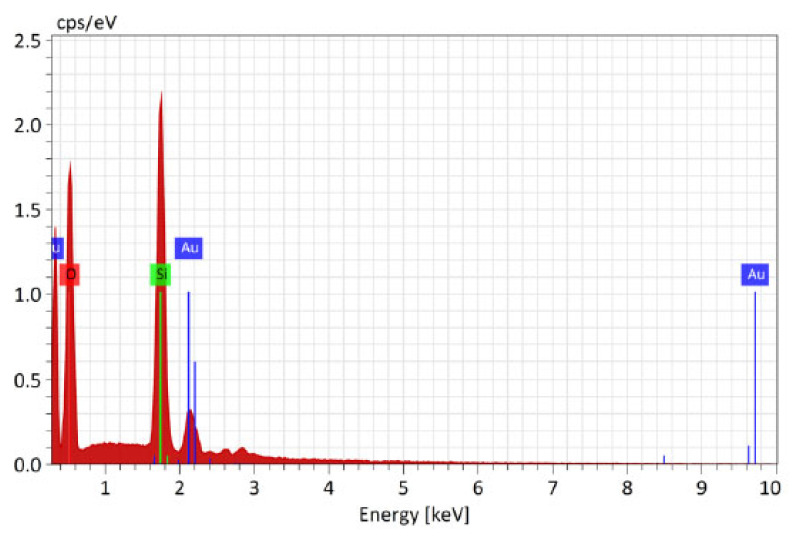
SEM-EDX spectra of surface of the sample modified with catalyst with detected Au nanoparticles.

These findings suggest that the samples of electrospun fibers after modification with antimicrobial coating containing Au nanoparticles retained their mechanical and chemical properties while simultaneously acquiring new antimicrobial coating properties through sol-gel coating with added Au nanoparticles on the sample surface (SEM-EDX micrograph, Figure 7, and SEM-EDX mapping with EDX elemental analysis, Figure 8 and Figure 9, Table 5). 

**Table 5:** Results of sample surface with detected Au, Si and O and their average surface mass ratio.

Element	At. No.	Netto	Mass [%]	Mass Norm. [%]	Atom [%]	Abs. Error [%]	Rel. Error [%]
O	8	32,074	26.28	44.46	64.42	1.01	3.86
Si	14	44,955	24.25	41.04	33.87	0.92	3.79
Au	79	7643	8.57	14.50	1.71	0.36	4.22
		Sum	59.10	100	100		

The interface in the structured organic–inorganic composites, prepared from the precursor (3-glycidyloxypropyl) trimethoxysilane during the sol-gel coating process, was characterized using NMR, SEM-EDX, FTIR, and Raman spectroscopy. The combined application of these methodologies provided valuable insights into the local chemical environments and dynamic heterogeneities occurring within the deep coating reaction. Thorough characterization of precursor materials can enhance the correlation between precursor properties, preparation processes, and the performance of the final product, ultimately yielding improved results.

#### 3.4.2. Results of EDX Investigation of Samples after the Modification without Catalyst Recorded by Bruker Nano GmbH Berlin, Germany (SEM Tescan Vega)

It was clear that the precursors used had dissolved and that the morphology of the films obtained was regular. The composite films were assessed by mapping the surface of samples. The results show that the particles in the film were almost all evenly distributed and were in the form of small particles in cases when the catalyst was used (Figure 8 and Figure 9), while particles in the film agglomerated unevenly in cases when no catalyst was applied.

**Figure 10:**
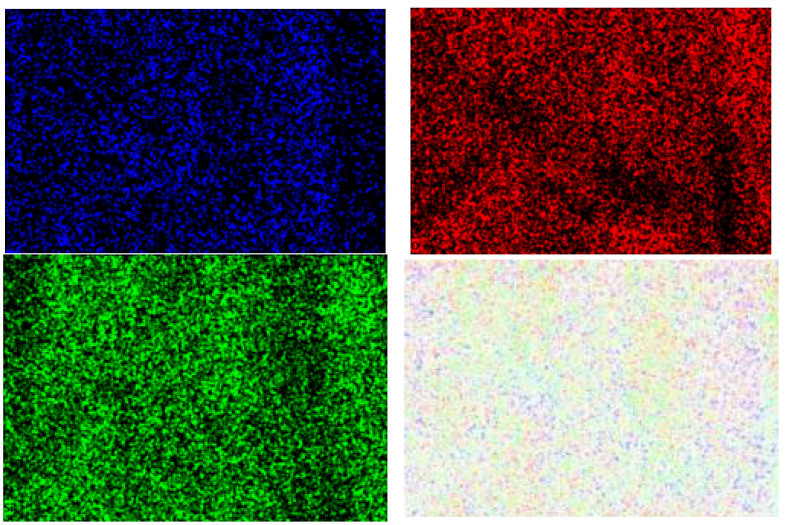
SEM-EDX mapping of surface sample after modification with the catalyst, with detected Au nanoparticles (blue), and O (red) and Si (green) elements.

**Figure 11:**
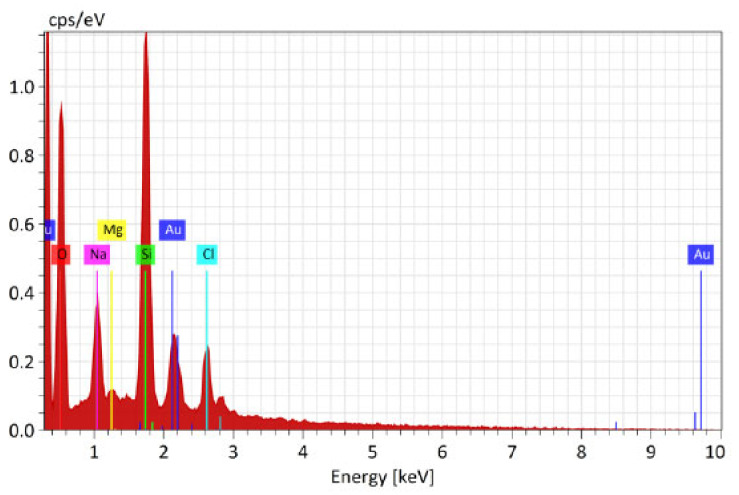
SEM-EDX spectra of surface of the sample modified without catalyst with detected Au nanoparticles, but also Sl, Na and Mg elements together with O and Si.

**Figure 12:**
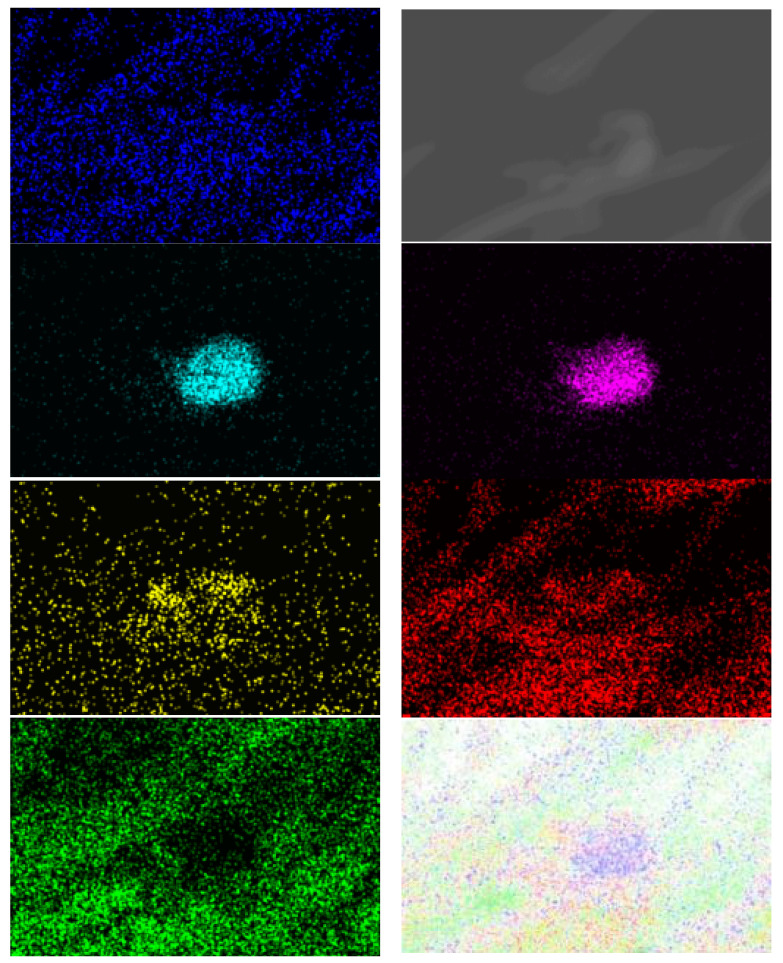
SEM-EDX mapping of surface sample after modification without the catalyst, with detected Au nanoparticles (blue), but also Cl (tirquise), Na (pink) and Mg (yellog) joined with O (red) and Si (green) elements.

**Figure 13:**
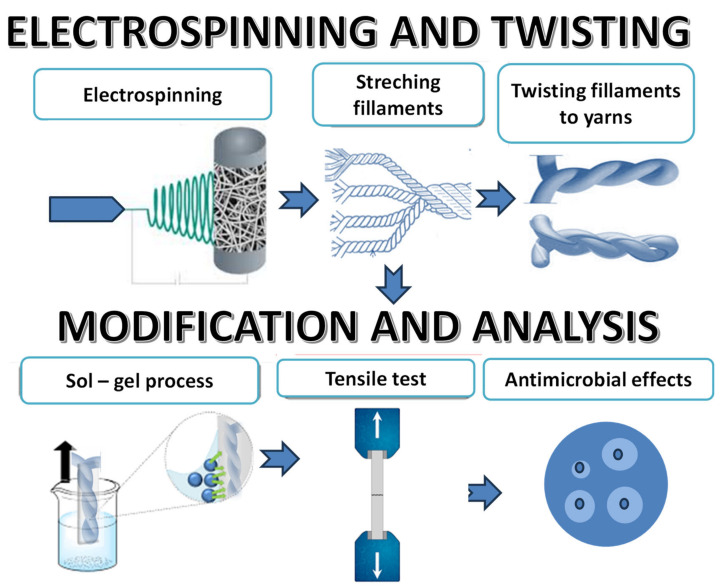
Schematic overview of the modification of electrospun fibers.

**Figure 14:**
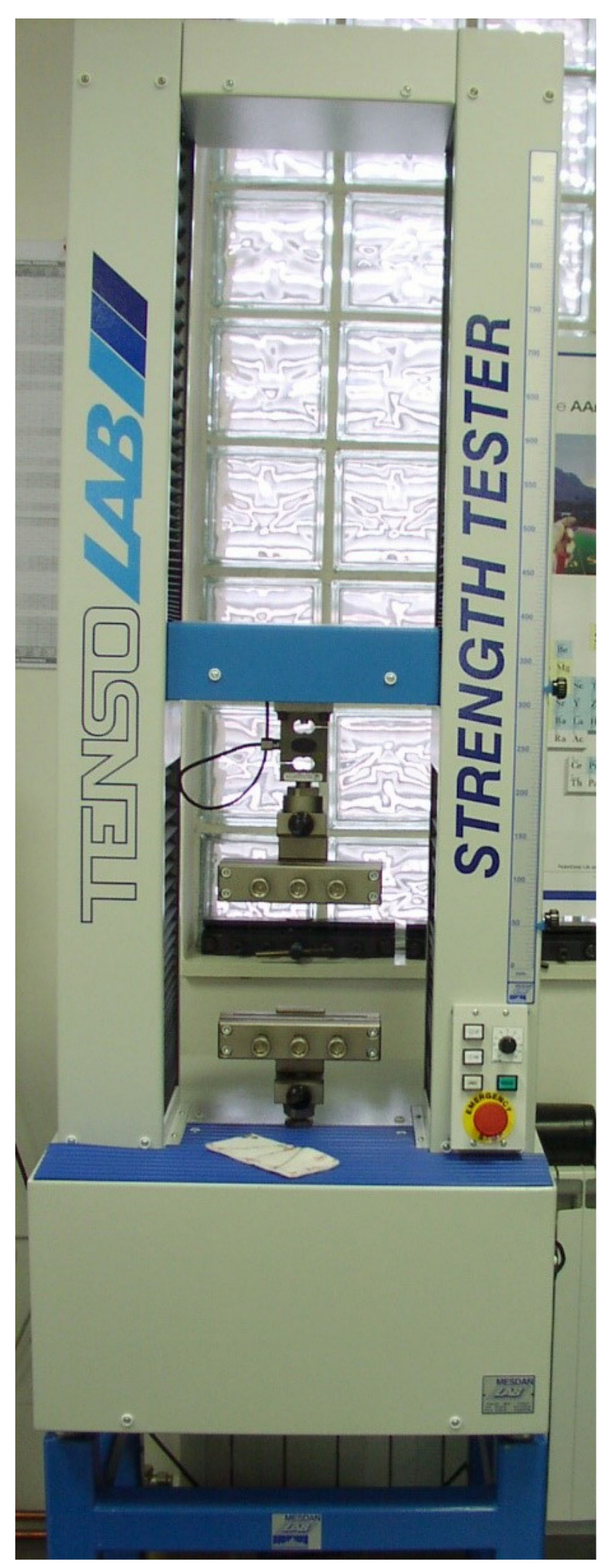
MesdanLab Strength Tester, S.p.A., Italy, Tensolab 3000, used for HRN EN ISO *2062:2010 Textiles—Yarns from packages—Determination of single-end breaking force and elongation at break using constant rate of extension (CRE) tester (ISO 2062:2009; EN ISO 2062:2009)* [55,56].

In addition, the mechanical properties were much worse after sample modification, but the usage of catalyst preserved some of the mechanical strength. More precisely, the results of single-end breaking force and elongation at break using a constant rate of extension (CRE) tester presented in Figure 5 show a decrease of 20 percent in breaking force and elongation at break, which is in agreement with the data presented in Table 2, Table 3 and Table 4. From this, it can be concluded that non-treated electrospun fibers can be applied without sol-gel modification in cases when mechanical properties are much more important than antibacterial effects. However, in cases when it is required to prevent the antimicrobial infection, the process with the usage of GLYMO as a precursor and HCl as a catalyst is advised. Without a catalyst, it is almost impossible to obtain a homogenous distribution of Au nanoparticles, as agglomeration of Na, Mg and Cl occurs (Figure 10, Figure 11, Figure 12, Figure 13 and Figure 14).

A major drawback was that, without the catalyst, a significantly lower amount of Au nanoparticles (10%) present in the surface of the materials was observed, as can be seen in Table 6.

**Table 6:** Results of sample surface with detected Au, Si and O and their average surface mass ratio.

Element	At. No.	Netto	Mass [%]	Mass Norm. [%]	Atom [%]	Abs. Error [%]	Rel. Error [%]
O	8	17,068	16.30	35.81	55.80	0.65	4.01
Si	14	23,441	14.51	31.86	28.29	0.57	3.91
Au	79	6381	7.58	16.65	2.11	0.32	4.19
Cl	17	4805	3.80	8.34	5.86	0.16	4.15
Na	11	5489	3.15	6.91	7.49	0.20	6.22
Mg	12	393	0.20	0.43	0.44	0.03	17.50
		Sum	45.54	100	100		

Tensile tests revealed that the mechanical characteristics of the electrospun filament yarns remained largely unaffected by the sol-gel process, showing no significant increase or decrease. This suggests that this treatment could serve as a viable approach for producing antimicrobial active implants. Furthermore, preliminary antimicrobial investigations indicated promising antimicrobial activity against MRSA and MSSA strains, although SEM images revealed a non-uniform distribution of nanoparticles and their tendency to agglomerate on PCA filaments.

Hence, future research efforts will focus on addressing these issues by incorporating additional homogenization steps, such as sonication or intensive stirring, to improve nanoparticle dispersion. Additionally, the reproducibility of the obtained results with the same nanoparticles, as well as with other agents approved by the Federal Drug Administration, will be thoroughly investigated.

The results demonstrate that sol-gel modification of electrospun filament yarns can be effectively achieved through dip coating methodology using GLYMO as a precursor, with or without a catalyst, and incorporating Au nanoparticles without compromising the integrity of the yarns or compromising their mechanical properties. Table 7 presents process parameters used in this research work.

**Table 7:** Process parameters of the treatments used in research.

Code	Parameters of Sol-Gel Process
A	GLYMO (3-glycidoxypropyltrimethoxysilane, 98%, Aldrich Chemicals, St. Louis, MO, USA) without catalysts! stochiometric ratio 1:3 20 °C; time of treatment: 1 h; dip-coating, 1 mm/s
B	GLYMO (3-glycidoxypropyltrimethoxysilane, 98%, Aldrich Chemicals) with catalysts, 0.1 mol/L HCl, 1.6 mol/L C_2_H_5_OH stochiometric ratio 1:1.5 20 °C; time of treatment: 1 h; dip-coating, 1 mm/s
**Active compounds**	
	Gold nanoparticles, 5 nm in diameter stabilized suspension in 0.1 mM PBS Product code: 4101784179, Lot MKCK 4830

The antimicrobial efficiency of Au nanoparticles that were applied on electrospun fibers and yarns was assessed on model microorganisms *Staphylococcus aureus*, *Escherichia coli*, and *Candida albicans*, and the results are presented in Table 8 and Table 9.

To ensure the accuracy and reliability of the method, Norfloxacine (10 µg/mL), Nistatine (1 mg/mL) and Gentamicine (10 µg/mL) were used as control standards to check the quality of the assay and the susceptibility of the bacterial strains. All tests were performed in quintuplicate, and the results showed that Au NPs showed significant antimicrobial activity against all three model microorganisms when testing by dilution methodology, while when investigating by diffusion methodology, the expected antimicrobial activity was not found (Table 7 and Table 8). This can be attributed to the mechanism of Au NPs antimicrobial activity.

We compared its effects to those of Ag NPs, which have a different mechanism of efficiency, and completely contradictory results were found—while Ag showed no antimicrobial efficiency, Au showed positive outcomes. Therefore, many additional tests are needed in order to determine the most appropriate combination of nanoparticles on the surface of electrospun yarns for medical purposes.

The unique physicochemical properties of gold nanoparticles (AuNPs) make them an appropriate component for increasing the conductivity of scaffolds to enhance the electrical signal transfer between neural cells [49]. The effect of Au NPs on the surface of Au-NPs-decorated scaffolds to promote neuronal differentiation and maturation was investigated by Baranes et al. [50]. The results showed that neurons cultivated on the gold nanoparticle scaffolds prefer axonal elongation over forming complex branching trees. The authors envisioned that such cellular constructs may be useful in the future as implantable cellular devices for repairing damaged neuronal tissues, such as the spinal cord.

Samadian et al. reported that they fabricated a 3D scaffold based on a poly (l-lactic acid) (PLLA)/Polycaprolactone (PCL)-matrix polymer containing gelatin nanofibers (GNFs) and gold nanoparticles (AuNPs) as the scaffold for bone-tissue-engineering application. Their in vitro studies showed that the highest concentration of AuNPs (160 ppm) induced toxicity and 80 ppm AuNPs exhibited the highest cell proliferation. The in vivo studies showed that PCL/PLLA/Gel/80ppmAuNPs induced the highest neo-bone formation, osteocyte in lacuna woven bone formation, and angiogenesis in the defect site. In conclusion, this study showed that the prepared scaffold exhibited suitable properties for bone tissue engineering in terms of porosity, pore size, mechanical properties, biocompatibility, and osteoconduction activities [51].

Matson et al. combined electrospun PCL with Au NPs and soy lecithin composite material for tissue-engineering applications [52,53,54] and proved that it is possible to successfully electrospin a lecithin, gold nanoparticle, and polycaprolactone scaffold for tissue-engineering applications. For this reason, future investigations should combine the results reported in the work with a step forward in the direction of direct introduction of Au NPs during the fabrication of the scaffolds. Through this step, not only might the antimicrobial activity be prolonged, it would also be more efficient on different microorganism strains.

The gold nanoparticles in colloid suspension were preliminary tested for their activity using standardized methods to determine their efficacy against various microbial strains. The tests performed included the minimum inhibitory concentration (MIC) assay and agar diffusion test. Firstly, the MIC assay determined the lowest concentration of gold nanoparticles required to inhibit visible microbial growth in liquid media, and through this, the MIC value provided a quantitative measure of the nanoparticles’ antimicrobial potency. Secondly, the agar diffusion test was performed in agar plates inoculated with microbial cultures, and the colloidal suspension of gold nanoparticles was introduced into the wells. The formation of inhibition zones around the wells indicated that the antimicrobial activity of the nanoparticles is strong for some concentrations.

The obtained results from the antimicrobial tests are presented in Table 7 and Table 8 and those demonstrate that the gold nanoparticles in colloidal suspension exhibited significant antimicrobial activity. The MIC assay revealed that even at low concentrations, the nanoparticles effectively inhibited microbial growth. The agar diffusion test further confirmed this, with clear inhibition zones observed around the wells containing the gold nanoparticle suspension. A key finding from those preliminary results was that Au nanoparticles tended to exhibit higher antimicrobial activity than Ag when observed with dilution tests, likely due to different mechanisms of their antimicrobial activity.

The antibacterial activity of AuNPs is not limited to ROS activity. It is revealed that AuNPs can also target the energy metabolism and transcription process of bacteria. uNPs are agglomerated on the bacterial surface and bound to the membrane protein due to the affinity of gold to the proteins. The formation of IB-AuNPs disrupts the bacterial membrane, and they are attached to cytoplasmic proteins with IB-AuNPs affinity, subsequently leading to the death of the bacterium. The mechanism discovered in this study is another example of the antibacterial activity of AuNPs, which is entirely independent of ROS creation [53]. Additionally, the stability of the colloidal suspension played a critical role in ensuring consistent and effective antimicrobial action, as the aggregation of nanoparticles could reduce their availability to interact with microbes. [54]

Figure 13 shows a graphical overview of the testing protocols that were applied after the electrospinning of the samples.

After chemical characterization, a well-established method was used for the determination of single-end breaking force and elongation at break using MesdanLab Strength Tester, S.p.A., Puegnago del Garda Italy, Tensolab 3000 (Figure 12), after which the antimicrobial investigation was performed.

## 4. Conclusions

In this work, we proposed the functionalization of continuous PCL yarns produced by electrospinning (see Figure 11) through a dip coating methodology, used for creating novel composite materials with Au nanoparticles in their surfaces. It was shown that the adhesion of gold nanoparticles on the samples could be improved by modifying the surface by using an acid catalyst, which leads to better mechanical properties, as well as to a homogenous and equal distribution of Au nanoparticles on the surface of electrospun samples. Research endeavors integrating AuNPs with yarns have unveiled promising prospects for the creation of antimicrobial materials tailored for healthcare and hygiene applications, serving as effective infection control measures and barrier materials with enhanced functionalities. However, further investigations are needed to enhance the antimicrobial efficacy of AuNPs on the electrospun samples. Therefore, we anticipate that the combined approach outlined here will yield antimicrobial materials suitable for hospital care applications, addressing both the challenges of surgical failure and antibiotic-resistant infections.

## Figures and Tables

**Table 8:** Diffusion methodology for determination of antimicrobial activity of Au nanoparticles.

	Inhibition Zone, mm			
Microorganism	Au, 10 nm 20 ppm	Norfloxacine 10 µg/mL	Nistatine 1 mg/mL	Gentaicine 10 µg/mL
*Staphylococcus aureus*	0	-	-	17
*Escherichia coli*	0	23	-	-
*Candida albicans*	0	-	21	-

**Table 9:** Dilution methodology for determination of antimicrobial activity of Au nanoparticles.

	Nanoparticles	
Microorganism	Ag, 10 nm 20 ppm	Au, 10 nm 20 ppm
*Staphylococcus aureus*	0	Enhanced activity
*Escherichia coli*	0	Enhanced activity
*Candida albicans*	0	Enhanced activity

## Data Availability

Data underpinning this work will be available upon request.
